# Positive day-to-day relationships between the cortisol and testosterone awakening responses in elite male athletes

**DOI:** 10.5114/biolsport.2025.148541

**Published:** 2025-04-14

**Authors:** Blair T Crewther, Benjamin G Serpell, Zbigniew Obmiński, James McLaren, Phillip Fourie, Christian J Cook

**Affiliations:** 1 School of Science and Technology, University of New England, Armidale, Australia; 2 Queensland University of Technology, Brisbane, Australia; 3 Institute of Sport – National Research Institute, Warsaw, Poland; 4 Richmond Tigers Football Club, Melbourne, Australia; 5 University of Canberra Research Institute for Sport and Exercise (UCRISE), University of Canberra, Canberra, Australia; 6 Geelong Cats Football Club, Geelong, Victoria, Australia; 7 Hamlyn Centre, Imperial College, London, UK

**Keywords:** Adrenals, Stress, Gonads, Recovery, Adaptation, Coupling

## Abstract

The cortisol awakening response (CAR) shows promise as a tool for tracking stress, recovery, and fatigue, although questions around CAR stability in elite sport exist. A functional cortisol link to testosterone and its awakening response (TAR) could also affect the CAR and endpoint outcomes, with stress as a moderating factor. To help resolve these issues, we investigated the day-to-day dynamics of the CAR and TAR in elite athletes and controls. Saliva samples (i.e., waking, waking+30 minutes) were taken from 29 male rugby players (mean age 26.4 years) on four consecutive days and 42 male controls (age 31.0 years) on two consecutive or non-consecutive days. Relative CAR_∆30_ and TAR_∆30_ were computed as a reactive change score. All of the hormonal variables showed poor short-term stability in elite athletes, with only slight improvements among controls. On average, a significant and similar rising CAR_∆30_ was seen in athletes (42.0%) and controls (27.0%), whilst the TAR_∆30_ declined similarly in both cohorts by -11.0% and -15.5%, respectively. In elite athletes only, the CAR_∆30_ and TAR_∆30_ were significantly and positively related, likewise for all other cortisol-to-testosterone variable comparisons (partial r = 0.19–0.45). In summary, substantial daily variation emerged in the relative CAR and TAR, especially in elite male athletes. However, only elite athletes presented positive CAR and TAR relationships that extended to their constituent components. These signals could represent a coordinated system to prepare for and respond to daily stressors in elite sport, which also offers a complex regulatory mechanism for controlling the CAR.

## INTRODUCTION

The hypothalamic-pituitary-adrenal (HPA) axis manages the release of cortisol, a hormone that exerts pleiotropic effects on the nervous system, muscles, and athletic performance [1]. The cortisol awakening response (CAR) is a specific HPA-axis feature with growing interest in sport and exercise [2]. The CAR, which is characterized by a 50–75% surge in cortisol that peaks 30–45 min after awakening [3], is thought to be relatively stable over time [3, 4] with a significant heritable contribution [5]. In different sport and exercise settings, the CAR has shown promise as a tool for assessing stress, recovery, and fatigue [6, 7, 8, 9, 10, 11], but is not yet considered a reliable clinical tool. One possible reason is that athletes, especially those involved in elite level sports, are exposed to a myriad of overlapping stressors [12], which can affect the stability of the CAR and any resultant trends or predictions arising from it.

A functional connection between the HPA axis and hormones of the hypothalamic-pituitary-gonadal (HPG) axis (e.g., testosterone) is another consideration. Both axes form part of a neuroendocrine system that regulates different training processes [1] and coordinates other physiological systems to prepare for, and adapt to, stress more generally [13, 14]. Like cortisol, testosterone also presents a reliable awakening response (TAR) that is under some genetic control [15, 16, 17, 18], although the TAR declines during the same post-awakening period. Interestingly, the post-awakening trajectories of cortisol and testosterone diverge, but cross-sectional studies report positive CAR and TAR relationships [16, 17, 18]. This pattern indicates that those individuals who presented a flatter (or rising) TAR also tended to possess a larger CAR [16]. To our knowledge, no longitudinal study has explored these positive linkages or specifically between day-to-day fluctuations in the CAR and TAR.

As a composite measure of hormone reactivity, the CAR is logically shaped by those components that comprise this feature–the waking and post-waking measures [19, 20]. Positive testosterone and cortisol relationships, at one or both sampling points, have been seen in healthy adults [15, 17, 21] and athletic populations [22]. These results are congruent with reports of positive within-person testosterone and cortisol relationships (or positive hormone coupling) across the day [21, 23, 24]. This includes positive effects of pulse-like testosterone and cortisol fluctuations on each other [25] that, at least conceptually, could apply to the TAR and CAR. Thus, an early-morning shift in testosterone concentration might influence cortisol availability, thereby affecting the CAR and its primary role in energy mobilization and/or regulation of emotional experiences [26]. Our argument is that this early-morning interplay is another manifestation of functional cross-talk between the HPG and HPA axes [13, 14], and one that is perhaps necessary to achieve these endpoint outcomes.

If stress promotes stronger or more persistent positive coupling between the HPA and HPG axes [24, 25], then any cortisol and testosterone relationships might be more marked in elite sport than in non-sport settings. A longitudinal study on professional male rugby players and male students examined this possibility [27]. The salivary cortisol and testosterone relationships (on basal measures) were positive in both groups, but somewhat stronger in male students. Some sampling bias was, however, evident with data collected at different times of the day (i.e., elite athletes at 0800–0900 hrs, students at 1330 hrs on average) and in cohorts recruited from different countries. Research targeting the post-awakening period is limited. One study monitored the CAR and TAR in climbers and controls [28], although it did not conduct a formal group comparison of the post-awakening responses. More longitudinal evidence, ideally using a robust case-control design, is lacking in the literature. This gap can be addressed by the daily CAR and TAR assessment of elite athletes and non-athletes and, in doing so, would add novel insight into the regulatory biology controlling the CAR.

To address the aforementioned issues or gaps, we investigated the day-to-day dynamics of the CAR and TAR in elite male athletes and male controls. Our main goals were to: (1) quantify the short-term stability of these features and their constituent elements in an elite sport (athletes) and non-sport (controls) environment; (2) compare the CAR and TAR profiles of each cohort; and (3) explore a broad set of CAR and TAR relationships. Our first hypothesis (H1) was that all hormonal variables would be less stable in the elite sport (vs. non-sport) setting. The second hypothesis (H2) was that the post-awakening cortisol and testosterone trajectories would match literature (i.e., rising CAR, falling TAR), but differ in relative magnitude between elite athletes and controls. Our final hypothesis (H3) was that elite athletes would present stronger positive CAR and TAR relationships, as well as stronger CAR or TAR relationships (also positive) with waking hormones than controls.

## MATERIALS AND METHODS

### Participants

This study used a hybrid strategy, combining three unpublished datasets (N = 71) for analysis. This number represents 29 elite male athletes (age = 26.4 (3.9) years, height = 186.0 (9.4) cm, and body mass = 104.0 (13.4) kg) playing professional rugby union and 42 healthy untrained men (age = 31.0 (9.6) years, height = 176.2 (14.1) cm, and body mass = 81.2 (20.3) kg) who served as controls. A study investigator (BS), who was part of the coaching staff, recruited the athletes. Two investigators (BS and BTC) recruited the controls (via email) from a local chess club and research institute. Our classification of “elites” was based on 10 years (or more) of specialized training and participation in the southern hemisphere’s strongest rugby union league. Exclusion criteria included a history of regular exercise (controls only), any injuries affecting normal training (athletes only), a diagnosed endocrine or sleep disorder, recent international travel across different time zones, and use of any banned substances. We obtained written informed consent prior to data collection. The Human Research Ethics Committee at the University of New England (number HE22-141) provided study approval.

### Study design

We employed a longitudinal, case-control design to test the study hypotheses. The elite-athlete cohort was assessed for the salivary CAR and TAR on four separate occasions (days) and the control group was assessed on two occasions (days). Thereafter, we examined the short-term stability of the CAR and TAR and their waking components, before comparing the hormonal profiles of each group after data aggregation across this study. Finally, we investigated the day-to-day relationships between these hormonal variables and whether they differed in a cohort-specific manner. Conceptually, this format of testing is consistent with ideas on CAR flexibility and well-being linkages [29], as well as within-person HPA and HPG coordination [27], based on normal daily fluctuations in the stated outcomes.

### Cohort assessments

The elite male athletes were assessed over four consecutive days (day 1, day 2, day 3, day 4) at a pre-season training camp. These athletes self-collected saliva samples in a dormitory at the training facility, where they were housed at this time. A study investigator (BS) provided a detailed explanation of when and how to collect these samples, and he was present at the training camp to ensure full compliance. No exercise was performed in the 48-hour period before day 1. In the week prior to initial testing, the monitored athletes completed six technical/tactical field sessions (~6–8 hours), a combat session (~1 hour), and four gym sessions (~4–5 hours). The field and gym sessions were considered high in intensity and volume of work. Physical activity prescribed during the four-day sampling block was a continuation of this high-volume and/or high-intensity training with no resting days.

**FIG. 1:**
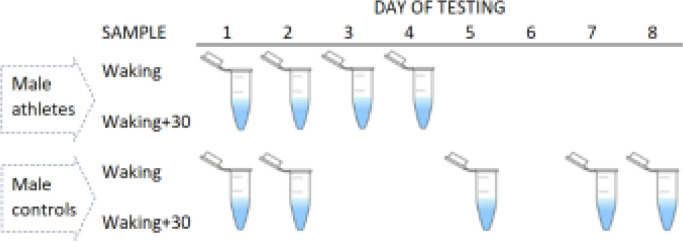
Summary of sample collections in each cohort. Elite male athletes were assessed over four consecutive days (4 tests per participant), whereas male controls were assessed less frequently (2 tests) and more intermittently on consecutive or non-consecutive days.

All men in the control group self-collected saliva samples at home, with a self-chosen starting date (day 1). Subsequent daily saliva collections were not fixed in time, as per the elite athlete cohort. The participants comprising this group provided samples on either two consecutive days (N = 14) or two non-consecutive days (N = 28) within a period of eight days. Overall, the participants reported saliva testing on day 2, day 5, day 7, or day 8, relative to day 1. To aid compliance, the participants received a detailed explanation of the sampling protocols (from BS and BTC), and were able to ask questions, with a text message or email sent prior to testing [19]. Figure 1 summarizes the sample timings in each group.

For each daily assessment, two saliva samples (~1 mL each) were taken using standard protocols in sport [8, 10, 28]. Briefly, this involved a passive drooling method, where saliva is allowed to pool at the bottom of the mouth and then eased into a 5-mL polypropylene tube before storage. The first sample was taken within three minutes of waking (range 0530–0730 hours) and the second sample was taken after a timed period of 30 minutes. All participants reported arising under spontaneous or externally woken (i.e., alarm) conditions, which appears to have little impact on the CAR [19]. We predicted that the TAR would be similarly robust to the mode of awakening. To eliminate any measurement bias, instructions were given to refrain from eating, drinking, smoking, or exercising before sampling [19]. The participants were also encouraged to get at least eight hours of sleep a night [19]. No direct measures of sleep quality or duration were available for this study.

### Hormone analyses

The saliva samples were stored in a -20° C freezer as soon as practicable after collection [19]. Upon study completion, the samples were picked up and delivered (on dry ice to ensure frozen samples on arrival) to the Stratech Scientific laboratory, Sydney. There, enzyme-linked immunoassay kits (Salimetrics LLC, USA) were used to determine cortisol and testosterone concentrations in saliva. The cortisol kit had a functional and analytical sensitivity limit of 0.018 µg/dL and 0.007 µg/dL, respectively. The functional and analytical sensitivity limits for the testosterone kit were 0.68 pg/mL and 0.458 pg/mL, respectively. Inter-assay variability on kit-supplied low and high controls in each plate, expressed as a coefficient of variation, did not exceed 5% on average. We tested all participants’ samples in the same plate to eliminate inter-assay variation in the measured hormones.

### Statistical analyses

To provide insight regarding the post-awakening hormone trajectories, the CAR and TAR were computed as measures of relative hormone reactivity [20, 30, 31]. To this end, we calculated a change score based on the log differences between sampling points [ln(waking+30) – ln(waking)], which we expressed here as the CAR_∆30_ and TAR_∆30_. This approach can normalize data distribution, and it accounts for non-uniformity bias arising from large individual or population differences in circulating hormones [32]. The log difference is also a close approximation of a percentage change (if multiplied by 100), at least when that difference is relatively small. Each hormonal feature was computed daily for each participant per test occasion. In total, we collected 200 measurements (elite athletes = 116, controls = 84) for each of the CAR_∆30_ and TAR_∆30_, as well as both waking hormonal metrics.

To assess the short-term stability of the CAR and TAR and their waking components, we calculated an intraclass correlation coefficient (ICC) for each variable in each cohort. We also calculated a second set of ICCs, adjusting for cohort differences in testing days. The ICCs were qualified as showing poor (< 0.50), moderate (0.50 to < 0.75), good (0.75 to < 0.90) or strong (0.90 to 1.00) stability [33]. To determine if the two cohorts differed hormonally, all variables were compared using a repeated measures analysis of variance. The data were modeled in a linear mixed-effects framework, specifying cohort (Athletes, Controls) as a fixed factor, the day of testing as a covariate, and a random intercept for each participant. In the event of a significant result, partial eta squared ($\eta_{\text{p}}^{2}$) and Cohen’s *d* were calculated as effect-size statistics. The $\eta_{\text{p}}^{2}$ values were interpreted as small (0.01 to 0.06), medium (0.06 to 0.14) or large (0.14+) effects. The *d* scores were interpreted as small (0.20 to < 0.50), medium (0.50 to < 0.80), large (0.80 to < 1.20) or very large (1.20+) effects [34]. The modeled results are shown as estimated marginal means with a 95% confidence limit (CL).

To identify any significant day-to-day hormonal relationships, we computed (in each group) a series of multilevel correlations [35] between the waking and post-awakening responses. We excluded the waking+30 data, as spurious results can arise when composite features (e.g., CAR, TAR) are modeled along with their underlying components, as is the case in normal regression. Multilevel correlations are equivalent to a linear mixed-effects model with a random intercept [35]. For each comparison, the effects of all other variables were controlled for (i.e., partial *r*). To interpret the partial *r* values, we applied the conventions of weak (*r* = 0.20 to < 0.40), moderate (*r* = 0.40 to < 0.60), strong (*r* = 0.60 to < 0.80) and very strong to perfect (*r* = 0.80 to 1.00) effects [36]. The *p* values were adjusted using a false-discovery controlling procedure. Finally, we tested for significant cohort differences in each partial correlation using Fisher’s r-to-z transformation (single-sided test) for independent samples. All statistical analyses were performed in R Studio (version 4.4.1) [37].

## RESULTS

The ICC’s for the cortisol variables were quite low (≤ 0.35) among elite male athletes, likewise for waking cortisol and relative CAR_∆30_ in male controls (ICCs ≤ 0.28), suggesting poor short-term stability for these outcomes (Table 1). The one exception was waking+30 cortisol in controls, which showed moderate stability (ICC = 0.67, adjusted ICC = 0.65). All of the testosterone variables exhibited poor stability in elite male athletes (ICCs ≤ 0.37), as did waking+30 testosterone and relative TAR_∆30_ among controls (ICCs ≤ 0.48). In the control group, waking testosterone showed improved (i.e., moderate) stability (ICC = 0.72, adjusted ICC = 0.73). In general, the calculated ICCs were lower in elite athletes than controls and lower for the CAR_∆30_ and TAR_∆30_ versus the waking and waking+30 variables.

**TABLE 1:** Stability statistics for each hormonal variable in elite male athletes and male controls.

Variables	Male athletes		Male controls	

	ICC	ICC^1^	ICC	ICC^1^
Cortisol				
Waking (ng/mL)	0.35	0.35	0.28	0.25
Waking+30 (ng/mL)	0.22	0.33	0.67	0.65
Relative CAR_∆30_ (log diffs)	0.04	0.15	0.23	0.22

Testosterone				
Waking (pg/mL)	0.18	0.23	0.72	0.73
Waking+30 (pg/mL)	0.36	0.37	0.48	0.47
Relative TAR_∆30_ (log diffs)	0.05	0.05	0.13	0.13

Key: ICC = intraclass correlation coefficient, ICC^1^ = covariate-adjusted intraclass correlation coefficient, CAR_∆30_ = cortisol awakening response, TAR_∆30_ = testosterone awakening response.

Table 2 displays the marginal means for waking and waking+30 hormones, once back-transformed into their original units. Our analyses yielded a significant cohort effect on waking+30 cortisol ($\eta_{\text{p}}^{2}$ = 0.09 or medium effect). The elite male athletes presented a higher cortisol concentration than male controls (*d* = 0.73) at this sample, which is a medium-sized effect. Comparisons of waking cortisol and relative CAR_∆30_ did not reveal any significant cohort effects. For testosterone, a significant cohort effect arose at the waking ($\eta_{\text{p}}^{2}$ = 0.08 or medium effect) and waking+30 ($\eta_{\text{p}}^{2}$ = 0.15 or large effect) samples. Elite male athletes presented a higher testosterone concentration at both time points (*d* = 0.65 and *d* = 0.92, respectively) than male controls, representing medium to large effect-size differences. The cohort effect on the relative TAR_∆30_ was non-significant. At the request of a reviewer, observed power (N = 1000 simulations, alpha = 0.05) was calculated for all significant (range from 66.4% to 92.5%) and non-significant (19.0% to 27.2%) models described above.

**TABLE 2:** Estimated marginal means for each hormonal variable in elite male athletes and male controls.

Variables	Male athletes		Male controls		

	Mean	95% CL	Mean	95% CL	p values
Cortisol					
Waking (ng/mL)	3.35	2.88, 3.83	3.03	2.62, 3.44	0.305
Waking+30 (ng/mL)	4.76^A^	4.20, 5.31	3.85	3.41, 4.28	0.011
Relative CAR_∆30_ (log diffs)	0.35	0.24, 0.47	0.24	0.12, 0.36	0.194

Testosterone					
Waking (pg/mL)	260^A^	239, 281	231	206, 255	0.019
Waking+30 (pg/mL)	223^A^	204, 242	183	168, 197	0.001
Relative TAR_∆30_ (log diffs)	-0.12	-0.18, -0.05	-0.17	-0.24, -0.09	0.299

Key: CAR_∆30_ = cortisol awakening response, TAR_∆30_ = testosterone awakening response. Note that multiplying the CAR_∆30_ and TAR_∆30_ by 100 approximates a percentage change, when the log differences are relatively small. ^A^Denotes a significant difference from male controls *p* < 0.05.

**FIG. 2:**
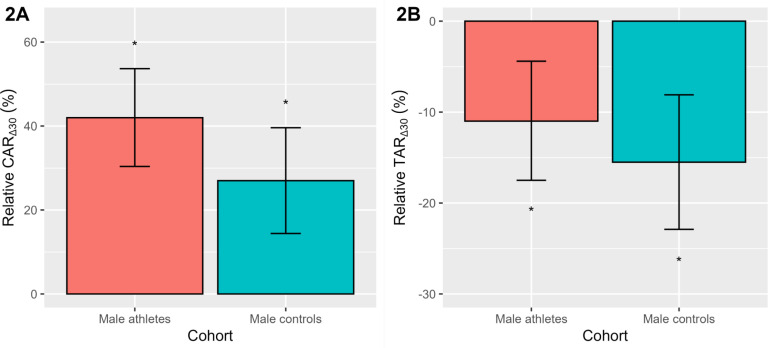
The relative awakening responses of cortisol (CAR_∆30_, 2A) and testosterone (TAR_∆30_, 2B) in elite male athletes and male controls. Data are presented as a percentage change with a 95% CL, after back-transformation of the log differences. *Denotes a significant percentage change *p* < 0.001.

To aid interpretation, we have plotted the relative CAR_∆30_ and TAR_∆30_ as a percentage change (Figure 2), after back transformation of the log differences. Significance testing of the estimated 95% CL was performed using the Wald statistic. As seen in Figure 2A, the study-averaged relative CAR_∆30_ was positive (*p* < 0.001) in elite male athletes (42.0%) and male controls (27.0%). The relative TAR_∆30_ (Figure 2B) was also similar across both cohorts, declining (*p* < 0.001) on average by -11.0% (elite athletes) and -15.5% (controls). As noted in Table 2, there were no significant cohort differences in the relative CAR_∆30_ or TAR_∆30_.

**FIG. 3:**
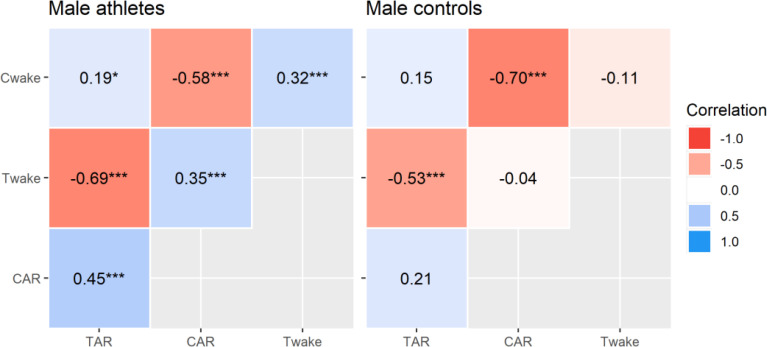
Multilevel partial correlations between selected hormonal variables in elite male athletes and male controls. Key: Cwake = waking cortisol, CAR = relative cortisol awakening response, Twake = waking testosterone, TAR = relative testosterone awakening response. Correlations are significant at *p < 0.05, **p < 0.01, ***p < 0.001.

Correlational analyses revealed significant *inter-hormonal* relationships that were limited to elite male athletes (Figure 3). This included positive correlations between waking testosterone and cortisol concentrations, waking cortisol and TAR_∆30_, waking testosterone and CAR_∆30_, and the CAR_∆30_ and TAR_∆30_. This means that each variable tended to rise and fall with the other, although the effect sizes were moderate at best. The *intra-hormonal* correlations were consistent in both groups and reflect established patterns in CAR research [19, 20]. That is, waking cortisol and CAR_∆30_ were negatively related, as were waking testosterone and TAR_∆30_ (all *p* < 0.001, moderate to strong effects). Hence, a higher waking concentration corresponded to a relatively smaller post-awakening response. Calculated power was strong for all significant correlations (94% to 100 %), except where *r* = 0.19 (54% power). The partial *r* values differed significantly (*p* ≤ 0.038) between elite athletes and controls, apart from the CAR_∆30_ (*p* = 0.080) and TAR_∆30_ (*p* = 0.389) relationships with waking cortisol.

**FIG. 4:**
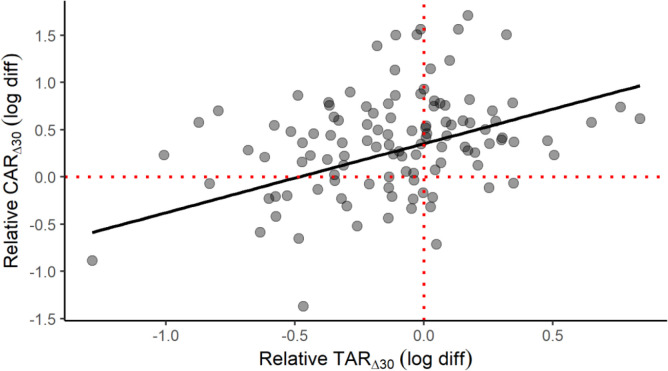
Scatterplot of the relative awakening responses of cortisol (CAR_∆30_) and testosterone (TAR_∆30_) in elite male athletes. The plotted slope is based on a linear mixed-effects model with selected covariates: see text for full details. The fitted model has a conditional R^2^ of 0.57 and a marginal R^2^ of 0.47.

A scatterplot is provided to help conceptualize the positive CAR_∆30_ and TAR_∆30_ relationship in elite male athletes (Figure 4), which is seemingly at odds with the divergent early-morning trajectories of cortisol and testosterone. This plot represents a fitted linear mixed-effects model with the TAR_∆30_ predicting the CAR_∆30_, waking cortisol and testosterone entered as covariates, and participant as a random effect. The effect of relative TAR_∆30_ (beta = 0.73, partial *r* = 0.45, *p* < 0.001) on relative CAR_∆30_ illustrates how this positive link can materialize. Waking cortisol (beta = -0.69, partial *r* = -0.62, *p* = 0.025) and waking testosterone (beta = 0.63, partial *r* = 0.35, *p* < 0.001) also contributed significantly to this outcome. Overall, the explanatory power of this model was deemed substantial, based on the combined random and fixed effects (shared variance of 57%) or fixed effects alone (47%). The model’s observed power (N = 1000 simulations, alpha = 0.05) was calculated at 99.9%.

## DISCUSSION

This study explored the daily dynamics of the CAR and TAR in elite male athletes and male controls. Most of the hormonal variables exhibited poor short-term stability, and, in support of H1, this instability was more pronounced in elite sport. Further testing revealed a rising CAR_∆30_ and declining TAR_∆30_ in elite athletes and controls. Contrary to H2, the relative changes observed were of similar magnitude in each cohort. The relative CAR_∆30_ and TAR_∆30_ also covaried (positively) in male athletes, as did all other cortisol-to-testosterone variable comparisons. In support of H3, these relationships were absent among male controls.

Our short-term estimates of stability for all hormonal variables were poor in elite athletes and slightly better among controls, but still moderate at best. In both cohorts, the waking and waking+30 variables were more stable than the relative CAR_∆30_ and TAR_∆30_. Another important interpretation of the low ICCs reported is that a substantial portion of the observed variance (27–95%) occurred at the day-to-day level. Some daily variability is to be expected, especially in elite sport, given exposure to stressors affecting hormone release, including training load or intensity [8, 9, 32] and prior day physical activity [6], with potential mediation by muscle damage [32], overtraining, and recovery status [7]. The absence of such stressors could explain why the control group presented relatively stronger ICCs. In literature, stability or reliability estimates for the CAR [3, 4, 31, 38] and TAR [30] can vary considerably (0.12–0.85), depending on factors like the target feature, number of sampling days, and sampling compliance. Research on elite military men employing stricter environmental control over two consecutive days [30, 31] found stronger reliability coefficients for the relative (%) CAR (*r* = 0.36) and TAR (*r* = 0.30). The TAR (*r* = 0.40) was also more reliable in a subset of compliant men, but not the CAR (*r* = 0.35).

On average, the elite athletes and controls presented a rising CAR_∆30_ (42.0% and 27.0% respectively) that generally parallels studies on athletic [7, 8, 9, 10, 22, 28, 32, 39, 40] and non-athletic cohorts [3, 4, 5]. Conversely, the average TAR_∆30_ declined in the elite athlete (-11.0%) and control (-15.5%) groups; trajectories that are consistent with data on athletes [28, 32, 39], elite military men [30], and non-athletes [15, 16, 17]. These results indicate some robustness in the CAR and TAR across populations and contexts. However, the timing and magnitude of these responses are affected by situational (e.g., overtraining status, recovery period, competition) and methodological factors (e.g., saliva vs. blood). We did not find any cohort difference in the relative CAR_∆30_ or TAR_∆30_. So whilst training for elite sport might promote homeostatic differences in testosterone and cortisol release [1, 32], as reported in Table 2, the relative post-awakening responses of these hormones are comparable to those of non-athletes after baseline corrections. A failure to account for this variance can lead to very different conclusions. As an example, when we expressed the CAR_∆30_ in absolute terms, the elite athletes presented a significantly larger cortisol response (mean = 1.33 [0.82, 1.83] ng/mL) than male controls (mean = 0.53 [0.04, 1.03] ng/mL).

All of the testosterone and cortisol variables (i.e., at waking, relative responses) were positively related in elite male athletes, but these relationships were notably absent in (and mostly significant from) the control group. Our finding of a positive CAR_∆30_ and TAR_∆30_ relationship in elite male athletes is consistent with reports on healthy [16, 17] and military men [18], although recent work on male judokas failed to find any significant CAR and TAR linkages [32]. The cited work does lack the rigor of a case-control design to establish whether a significant relationship in one group differs from another. The specificity of our findings could be adaptive to meet the daily stressors, both physical and psychological, of elite sport. The primary CAR functions are to mobilize resources to meet energy demands and counter-regulate adverse prior-day emotional experiences [26]. Given the speculated roles of the TAR when asleep (e.g., muscle anabolism) and awake (e.g., behavior and social interactions) [16], both signals could form a complementary system to ensure adaptive responding, according to situational and contextual cues that differ from one day to the next. These actions could be achieved via cross-talk between the HPG and HPA systems at each axis level [14].

The longitudinal design and case-control comparisons are strengths of this study. Advancing hormone-coupling research [18, 21, 23, 24, 25, 27], we also discovered that these positive testosterone and cortisol relationships apply to early-morning HPA and HPG activity (i.e., CAR-to-TAR, waking cortisol-to-TAR, waking testosterone-to-CAR), and are contingent on training status. This interplay could help reconcile mixed findings regarding CAR use in sport [2, 8, 9, 10, 22, 39, 40, 41]. One example is a flatter-than-expected CAR before a competition [10], which may arise from a lower waking testosterone concentration and/or a steeper decline in the TAR. In fact, the regression results indicated that up to 57% of the relative CAR_∆30_ in elite athletes was predicted by waking testosterone and relative TAR_∆30_, as well as waking cortisol level. Similarly, if the TAR and CAR correlate well, then any physiological prediction could be obscured when one feature is examined independently of the other. Expanding on these perspectives, it is also possible that the outcomes of stress, fatigue, and recovery might manifest, hormonally, in highly nuanced ways. To illustrate this point, a high-intensity training block in male judokas simultaneously suppressed the CAR (21%, 8%) and attenuated the TAR (-7%, -13%) at +30 and +60 minutes, respectively, compared to a light training block (CAR = 36%, 22%; TAR = -11%, -15%) [32].

These authors do acknowledge some shortcomings in this study. For instance, the two-point sampling schedule was chosen to improve compliance in sport, but may not capture the peak CAR [3], where a minimum of three samples (0, 30, and 45 minutes) are recommended [19]. Even so, the CAR and TAR in both cohorts were consistent with the literature. Adherence to the sampling protocols was also assumed, because we lacked the resources to conduct a sleep trial for verification. Even the self-reporting of adherence, as per this and other studies, is a potential source of error in CAR assessment [42]. To counter this, all participants were volunteers and naturally motivated to collect samples, as required, with verbal and written instructions given to ensure accurate timings and reliable collections. Due to the hybrid approach, sampling differences between athletes and controls also exist (e.g., sample size, number of testing days, consecutive vs. non-consecutive days). Therefore, one should exercise caution when interpreting any cohort effects (or lack thereof). Other validating datasets, addressing one or more of these limitations, are needed to derive more robust guidelines for CAR and TAR analysis and interpretation in sport and exercise.

## CONCLUSIONS

The relative CAR and TAR varied substantially each day in an elite sport setting, as did their underlying components, with only slight improvements in a non-sport (controls) setting. Despite this, elite athletes showed a positive relationship between the CAR and TAR that extended to their constituent elements. These relationships were notably absent in male controls. Accordingly, these signals could represent a coordinated system to prepare for and respond to daily stressors in elite sport, which could also affect the CAR and it’s predictive utility in sport and exercise via a complex regulatory mechanism.

## Data Availability

Due to ethical restrictions, the data collected is not publicly available.
